# Organelle-specific isoenzymes of plant V-ATPase as revealed by *in vivo*-FRET analysis

**DOI:** 10.1186/1471-2121-9-28

**Published:** 2008-05-28

**Authors:** Thorsten Seidel, Daniel Schnitzer, Dortje Golldack, Markus Sauer, Karl-Josef Dietz

**Affiliations:** 1 Department of Biochemistry and Physiology of Plants, W5, University of Bielefeld, 33501 Bielefeld, Germany; 2 Department of Laser Physics, D3, University of Bielefeld, 33501 Bielefeld, Germany

## Abstract

**Background:**

The V-ATPase (VHA) is a protein complex of 13 different VHA-subunits. It functions as an ATP driven rotary-motor that electrogenically translocates H^+ ^into endomembrane compartments. In *Arabidopsis thaliana *V-ATPase is encoded by 23 genes posing the question of specific versus redundant function of multigene encoded isoforms.

**Results:**

The transmembrane topology and stoichiometry of the proteolipid VHA-c" as well as the stoichiometry of the membrane integral subunit VHA-e within the V-ATPase complex were investigated by *in vivo *fluorescence resonance energy transfer (FRET). VHA-c", VHA-e1 and VHA-e2, VHA-a, VHA-c3, truncated variants of VHA-c3 and a chimeric VHA-c/VHA-c" hybrid were fused to cyan (CFP) and yellow fluorescent protein (YFP), respectively. The constructs were employed for transfection experiments with *Arabidopsis thaliana *mesophyll protoplasts. Subcellular localization and FRET analysis by confocal laser scanning microscopy (CLSM) demonstrated that (i.) the N- and C-termini of VHA-c" are localised in the vacuolar lumen, (ii.) one copy of VHA-c" is present within the VHA-complex, and (iii.) VHA-c" is localised at the ER and associated Golgi bodies. (iv.) A similar localisation was observed for VHA-e2, whereas (v.) the subcellular localisation of VHA-e1 indicated the *trans *Golgi network (TGN)-specifity of this subunit.

**Conclusion:**

The plant proteolipid ring is a highly flexible protein subcomplex, tolerating the incorporation of truncated and hybrid proteolipid subunits, respectively. Whereas the membrane integral subunit VHA-e is present in two copies within the complex, the proteolipid subunit VHA-c" takes part in complex formation with only one copy. However, neither VHA-c" isoform 1 nor any of the two VHA-e isoforms were identified at the tonoplast. This suggest a function in endomembrane specific VHA-assembly or targeting rather than proton transport.

## Background

The vacuolar H^+^- ATPase is a 700 kDa protein complex essential for all eukaryotes. The translocation of protons energised by the hydrolysis of ATP drives secondary transport across endomembranes generating a proton motive force [1,2]. This emphasises its importance for the maintenance of the intracellular pH and in ion homeostasis [3]. In addition, the V-ATPase is essential for sorting proteins within the secretory pathway [4], cell elongation in conjunction with plant development [5-7] and was postulated to be a key player in membrane fusion events in *S. cerevisiae *[8].

The V-ATPase is a bidomain enzyme complex comprising the cytosolic V_1 _sector and the transmembrane V_0 _sector. Both to be considered two functionally coupled subenzymes [9,10]. The chemical energy from ATP-hydrolysis within the catalytic head of the V_1_-domain is transduced into the rotation of the membrane-integral proteolipid ring that transports protons across endomembranes [11]. The ATP-hydrolysing hexameric head of alternatingly arranged subunits VHA-A and VHA-B forms the core complex of the V_1 _sector, while the proton-translocating sector V_0 _is dominated by a ring of most likely six proton-binding proteolipid subunits [12]. The proteolipid ring and the membrane integral portion of the 100 kDa subunit VHA-a catalyse the proton translocation across the membrane [10,13]. The proteolipid subunits of the V-ATPase are homologous to subunit c of the F-ATPsynthases and evolved by fused gene duplication from a common ancestor [14-16]. Accordingly, the subunit c of F-ATPsynthase is characterised by two transmembrane helices, whereas VHA-c possesses four transmembrane segments with only one proton binding site conserved at the fourth helix [14]. Though VHA-c" maintained two conserved glutamate residues during evolution which are located at helices 3 and 5, only the first one is functional [17]. It is due to structural peculiarities like the different positions of the catalytic glutamate residues, the low level of identity between VHA-c and VHA-c" and the presence of an additional helical segment in VHA-c" that the transmembrane topology is still subject of controversial discussion. Particularly, the hydrophobicity of the first helix remains unclear leading to topological predictions with either four or five transmembrane segments [18-21]. Furthermore, the helix 1 of the yeast VHA-c" was demonstrated to be essential for VHA-activity [22] whereas Nishi et al. (2003) [23] reported its insignificance for proton transport. However, this helix 1 is missing in plants and yeast. Δ*vma16*- (c"-) mutants were successfully complemented with Arabidopsis AtVHA-c" [18].

The small hydrophobic subunit VHA-e was first identified in bovine brain ATPase [24]. Recently the VHA-e homologue Vma9p was identified in yeast [25]. Two unexplored VHA-e isoforms exist in *A. thaliana *[12]. Current knowledge on VHA-e is restricted to its importance in V-ATPase assembly and function in yeast [25,26].

In recent years the orientation of the C-terminus of Vma16p was investigated intensively by exploring the accessibility of fused protein tags with tag-specific antibodies or proteolysis in the presence or absence of detergents, respectively [18-21].

To address these open question with an alternative technique, we applied *in vivo *FRET measurements to analyse the orientation of the C-terminus of VHA-c" relative to well characterised proteins like VHA-c and VHA-a. The FRET results demonstrate a high structural similarity of VHA-c" and VHA-c. Additional FRET-experiments indicate the presence of more than one VHA-e copy in the VHA-complex. VHA-c" and VHA-e isoform 1 assemble in distinct complexes with different subcellular localisation, whereas VHA-c" and VHA-e isoform 2 are incorporated in the same complex.

## Results

### *In silico *sequence analysis

**Figure 1:**
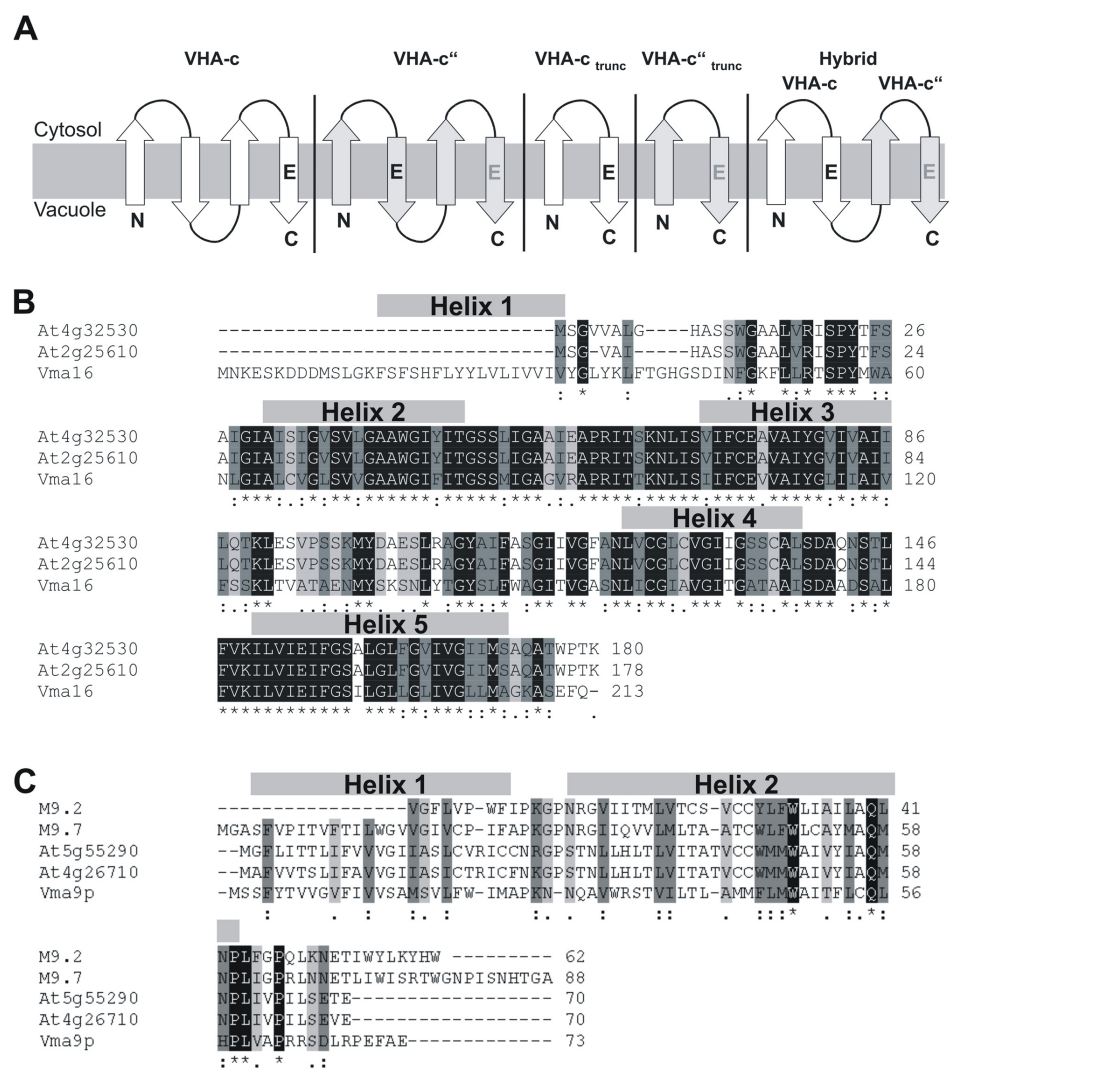
**Topology of proteolipid constructs and alignment of the VHA-c" and VHA-e amino acid sequences**. **(A) **Transmembrane topology and localisation of conserved glutamate residues in various proteolipid isoforms or synthetic constructs, namely VHA-c3, VHA-c", VHA-c_trunc_, VHA-c"_trunc _and hybrid proteolipid subunit. The functional Glu is highlighted in black, nonfunctional in grey. (B) VHA-c" sequences from *S. cerevisiae *(Vma16, Q91V37) and *A. thaliana *(VHA-c"1: At4g32530, CAB79970; VHA-c"2: At2g25610, NP_180132). **(C) **VHA-e sequences from *S. cerevisiae *(Vma9p, NP_958835), *Bos taurus *(M9.2, CAA75570), *Manduca sexta *(M9.7, CAA06822) and both VHA-e isoforms in *A. thaliana *(isoform 1: At5g55290, NP_568823; isoform 2: At4g26710, CAB79526) were compared. The sequences were obtained from the NCBI database and aligned using the ClustalW program. Transmembrane segments were predicted using HMMTOP and marked by bars. Identical amino acid residues are marked by (*) and highlighted in black, similar residues by (:).

Two isoforms of VHA-c" were identified in the *A. thaliana *genome [12]. The isoforms At2g25610 (VHA-c"2) and At4g32530 (VHA-c"1) are identical except of three amino acids at the N-terminus. Compared to the corresponding yeast proteolipid Vma16p plant VHA-c" lacks the first transmembrane helix resulting in a protein consisting of 146 aminoacids instead of 180. The remaining transmembrane segments of VHA-c" are highly conserved: 43 out of 146 aa are similar and 90 identical among the analysed sequences (Fig. 1B). The VHA-e amino acid sequences from *A. thaliana*, *B. taurus*, *S. cerevisiae *and *M. sexta *are identical to 17–32%, and the identity between both *A. thaliana *isoforms is 85%. Two transmembrane regions were identified comprised of aa 2–24 and 30–59 of AtVHA-e1 (Fig. 1C). To compare functional elements in VHA-c and VHA-c", a hybrid proteolipid (Fig. 1A) was constructed consisting of VHA-c helices 3–4 and VHA-c" helices 3–4. The variant is characterised by the presence of four predicted transmembrane segments. This helix arrangement matches the suggested ancestral topology of VHA-c- and VHA-c"-subunits. The molecular mass of the hybrid protein as calculated with Protparam was 17,195 Da as compared to 16,571 Da of VHA-c and 18,374 Da of VHA-c", and the isoelectric point IP was 7.6 which was similar to that of VHA-c" (7.8).

### Transcript analysis of proteolipid subunits

**Figure 2:**
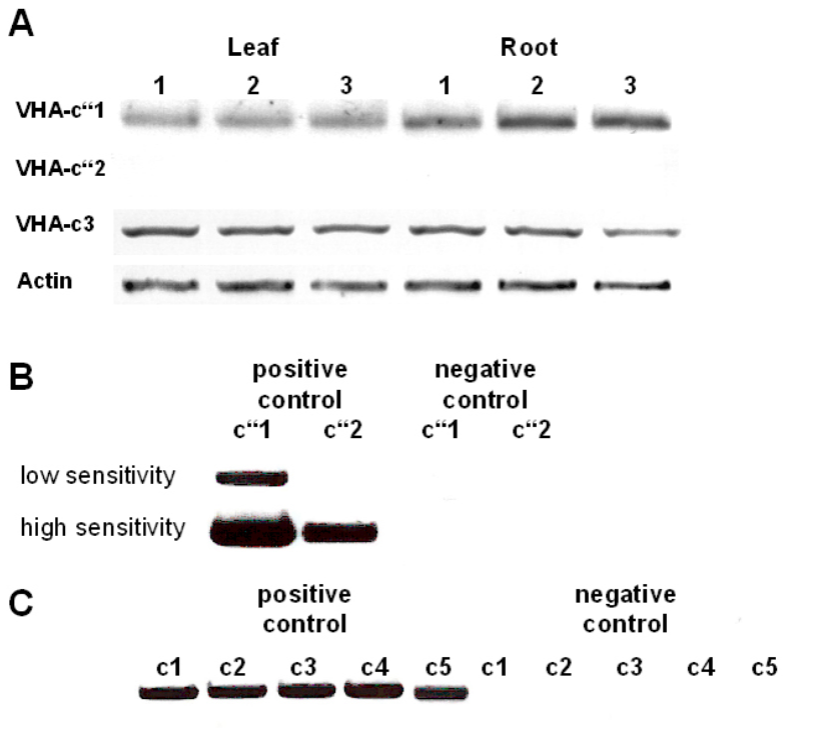
**Transcript quantification of VHA-c and VHA-c" isoforms**. (A) The transcript levels of VHA-c3 and VHA-c" isoforms were analysed in *A. thaliana *root and leaf tissue by semiquantitative RT-PCR in the linear range of amplification. Each experiment was performed three times as indicated with 1–3. Actin served as normalisation standard. (B) VHA-c"2 transcript was observed when images of ethidium bromide-stained agarose gels were obtained with increased exposure time (positive control). Using water as template for RT-PCR a PCR-product was not detectable neither for VHA-c"1 nor for VHA-c"2 (negative control). (C) Transcripts of the five VHA-c isoforms were detectable in leaf tissue (positive control), the negative controls using water as templates did not yield the PCR-product (negative control).

Transcript analysis of VHA-c isoforms by RT-PCR revealed the preferential presence of VHA-c2 and scarcely detected VHA-c1 in either roots or leaves of *A. thaliana *(data not shown). VHA-c3 transcript was detected in leaf tissue (Fig. 2A, C), although VHA-c3 was described as being expressed in a rather root specific manner before [27]. Our results are in line with a recent proteomics approach where VHA-c3 was identified at the tonoplast of *A. thaliana *leaf tissue [28]. The analysis of VHA-c" isoforms in *A. thaliana *revealed an almost exclusive expression of VHA-c"1 with only marginal expression of VHA-c"2 (Fig. 2A, B). Water as template served as a negative control in additional RT-PCRs and amplification products were absent as expected. These data supported the decision to subsequently focus the study on VHA-c"1 and to use VHA-c3 as reference in mesophyll protoplasts, except of the study on the subcellular localisation which also included VHA-c"2.

### Subcellular localisation

A set of genetic fusions between fluorescent proteins and VHA-subunits was produced in order to investigate the subcellular distribution of the proteolipid subunits as well as of the VHA-e isoforms. Besides full length constructs of VHA-c3, VHA-c"1, VHA-c"2, VHA-e1 and VHA-e2 truncated variants of VHA-c3 and VHA-c"1 were generated. The truncated variants comprised only the third and fourth helices of the proteolipid subunits VHA-c3 and VHA-c"1. Whereas the full length VHA-subunits and the truncated VHA-c were fused to the N-terminus of the fluorescent protein, the truncated VHA-c" was fused to the C-terminus of CFP and YFP, respectively. Furthermore, helices three and four of VHA-c and helices three and four were linked and fused to a fluorescent protein. This construct was denominated hybrid proteolipid subunit. The YFP fusion proteins were transiently expressed in *A. thaliana *mesophyll protoplasts. Fluorescence of V_0_-subunit constructs was observed at their corresponding destination endomembrane compartments within 3–4 hours. In the subsequent hours no indications were found for further alterations of the detected subcellular localisation, e.g. due to significant protein expression and accumulation.

**Figure 3:**
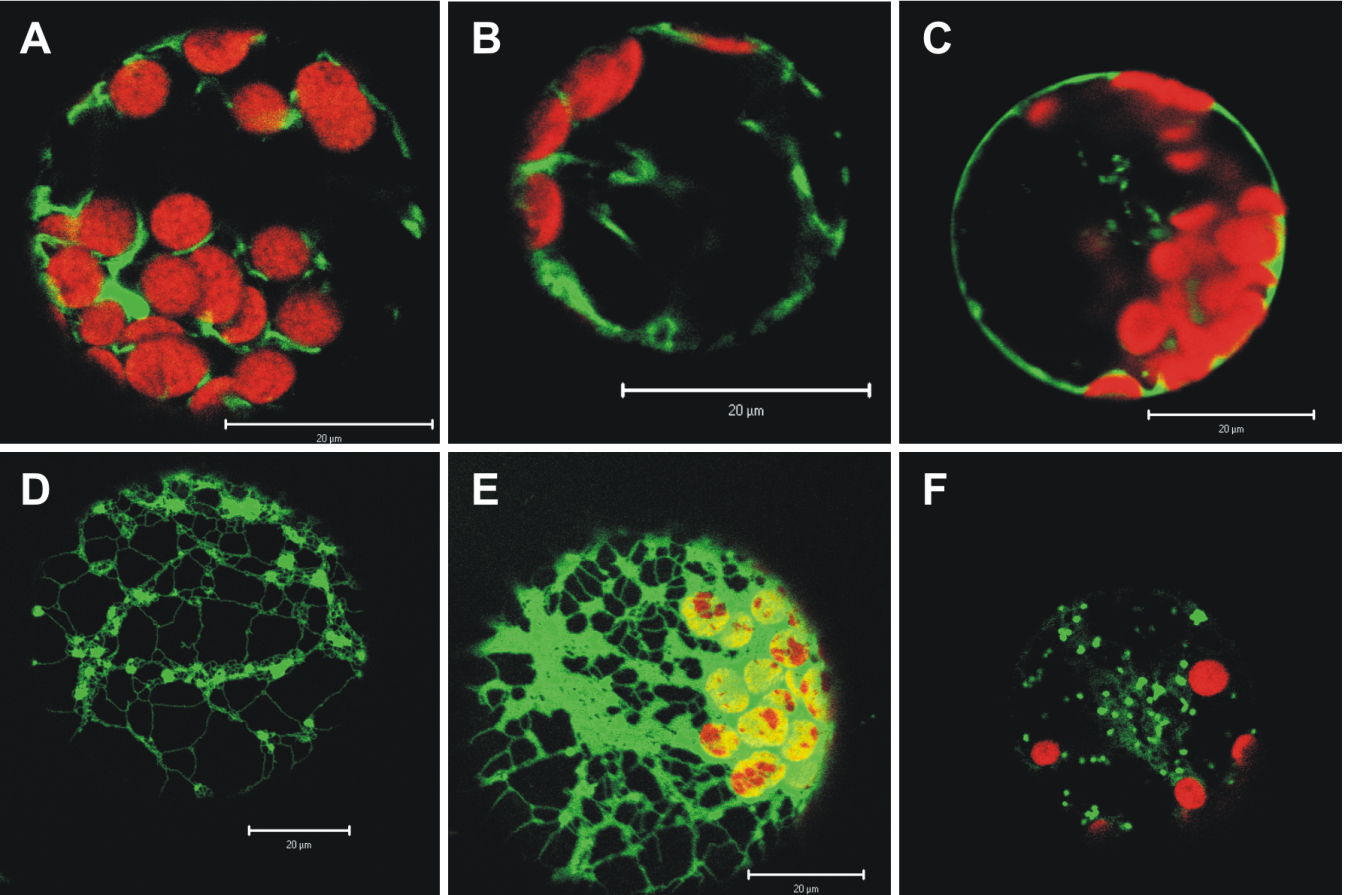
**Subcellular localisation of V_O_-subunits and proteolipid constructs**. VHA-c" (A, D), truncated VHA-c" (B), the VHA-c/VHA-c" hybrid proteolipid subunit (C) as well as VHA-e isoforms (E, F) were genetically fused to YFP and transiently expressed in Arabidopsis mesophyll protoplasts. The YFP fluorescence as shown in green was excited with 514 nm excitation and detected in the range of 530–600 nm by confocal microscopy. Chlorophyll autofluorescence is displayed in red and was detected in the range of 650–700 nm with 458 nm excitation. A, D: VHA-c"1. B: VHA-c"_trunc_. C: hybrid proteolipid. E: VHA-e2. F: VHA-e1. Scale bars represent 20 μm.

Analysis of VHA-c" revealed a distribution of both VHA-c" isoforms restricted to parts of the endomembrane system. Thus, fusion protein was detected in the ER-network and associated globular structures, but was absent from the tonoplast (Fig. 3). The truncated VHA-c"1 behaved in a similar manner. In contrast, the hybrid proteolipid subunit was localised at the tonoplast. The localisation pattern was similar to that previously reported for VHA-c and VHA-E [29]. The VHA-e isoforms revealed a distinct subcellular localisation. The VHA-e2-fusion protein gave a pattern alike that of VHA-c", while the VHA-e1 accumulated to low levels in the ER (Fig. 3). This particular image resembled that from previous observations for the TGN-specific subunit VHA-a1 [30].

### Stoichiometry of VHA-e and VHA-c"

**Figure 4:**
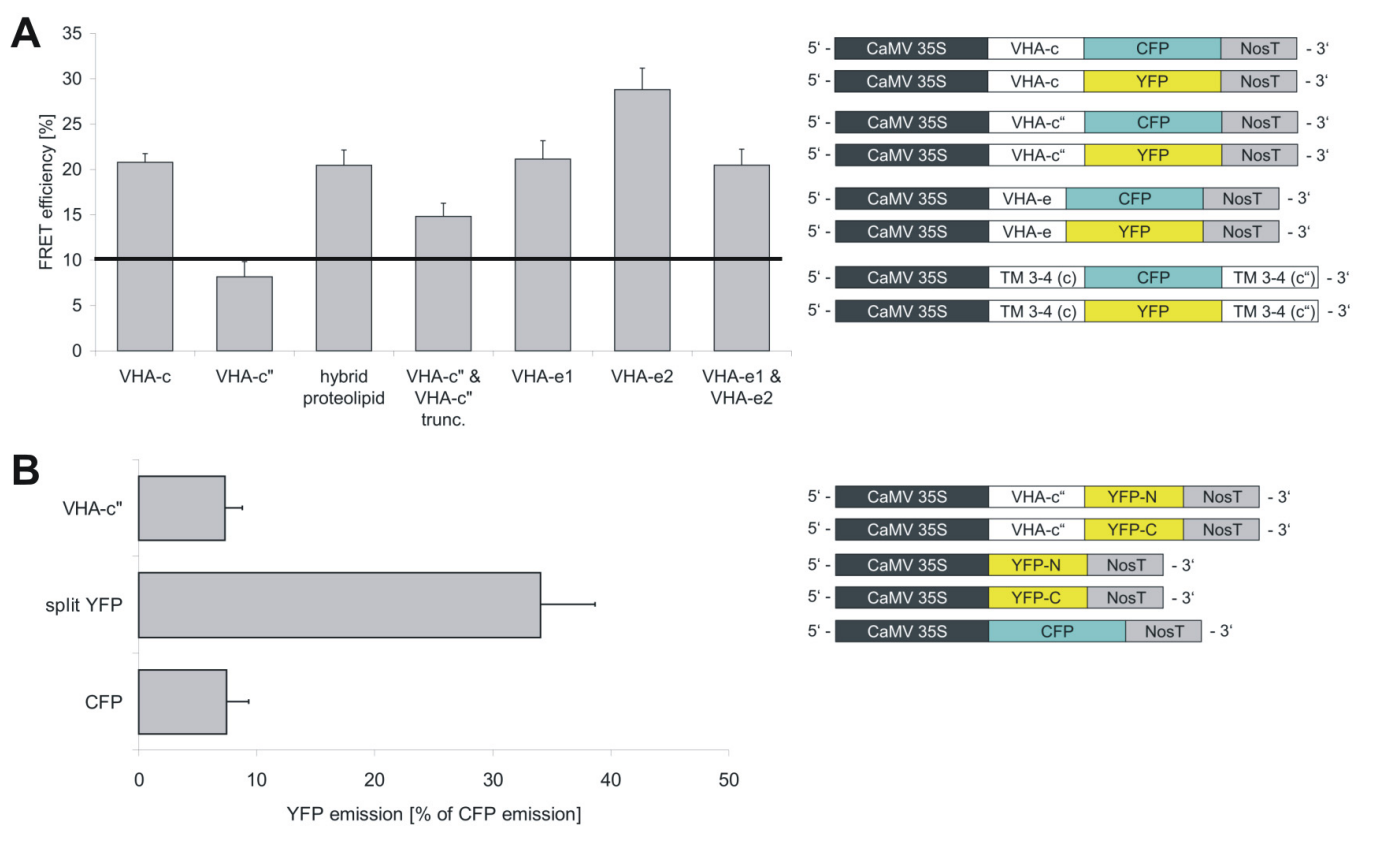
**Dimer formation of proteolipid constructs**. (A) Mesophyll protoplasts of *A. thaliana *were co-transfected with proteolipid subunits fused to CFP and YFP, respectively. A fusion protein of the transmembrane segments 3–4 of VHA-c and helices 3–4 of VHA-c"1 was engineered and is refered to as hybrid proteolipid. Observation of an energy transfer > 10% is taken as tentative indicator for the presence of more than one copy of the examined subunit in the complex. The data represent the mean average ± SE (VHA-c: n = 80; VHA-c": n = 71; hybrid proteolipid: n = 121; VHA-c"&VHA-c"_trunc_: n = 59; VHA-e1: n = 85; VHA-e2: n = 50; VHA-e1&VHA-e2: n = 78). (B) Bimolecular fluorescence complementation approach using VHA-c"-YFP-N-terminus and VHA-c"-YFP-C-terminus (VHA-c", n = 53). The BiFC-constructs and 35S-CFP-NosT were cotransfected. The YFP-fluorescence was normalised to CFP-fluorescence. YFP-formation of fluorophore fragments driven by the CaMV35S-promoter served as control for cytosolic self-assembly of the fluorophore (split YFP, n = 61) and expression of CFP under control of the CaMV35S-promoter served as background control (CFP, n = 25). The data represent the mean average ± SE.

To address the question whether one or more copies of VHA-c"1 and VHA-e1 assemble within the complex, protoplasts were co-transfected with two vectors each of the VHA-subunits fused to CFP or YFP and analysed for FRET-efficiency between the reporters *in vivo*. An increase in energy transfer efficiency was expected for those subunits that are known to be incorporated into the complex with several copies like VHA-c. Indeed, VHA-c showed a rate of energy transfer as high as 20.8%, whereas VHA-c" yielded only 8.2% indicating its presence as monomer in the complex (Fig. 4). No significant difference was identified by Student's T-test (p = 0.244) between the VHA-c"-CFP/VHA-c"-YFP- and the VHA-e1-CFP/VHA-c"-YFP-dataset, which serves as negative control. A FRET value of 8–10% was defined in earlier studies to mark the background level in the absence of structural interaction-based FRET due to the low signal intensity in the FRET-channel [29,31]. Despite the background level, the low signal intensity is also affected by the variability of CFP- and YFP reference measurements. Furthermore, the 10% FRET efficiency corresponds to a distance of approximately 7.2 nm and hence exceeds the diameter of the proteolipid ring, which was estimated to be 6–6.5 nm [32]. The FRET-data based conclusion on a single VHA-c" subunit was confirmed by a bimolecular fluorescence complementation approach. Cotransfection with two VHA-c"-constructs fused either to the C- or N-terminal fragment of YFP failed in functional fluorophore formation. In a converse manner, the control with cotransfected constructs coding solely for complementary N- and C-terminal fragments of YFP in the cytosol displayed a higher level of fluorophore formation. Interestingly, coexpression of VHA-c"-YFP and its truncated counterpart VHA-c"_trunc _fused to CFP revealed a FRET efficiency of 14.8%, thus indicating the co-existence of both proteins in single V-ATPase complexes. Measurements involving VHA-e fused to CFP and YFP, respectively, indicated the presence of more than one copy of VHA-e in the complex. The observed FRET efficiency was in the range of 20–25% for the combinations of VHA-e1/e1, VHA-e2/e2 and VHA-e1/e2. Apparently, a significant preference for isoform-specific interactions does not exist for the VHA-e1/e2 isoforms (Fig. 4) [see Additional files 1, 2, 3].

### FRET between transmembrane VHA-proteolipid subunits

**Figure 5:**
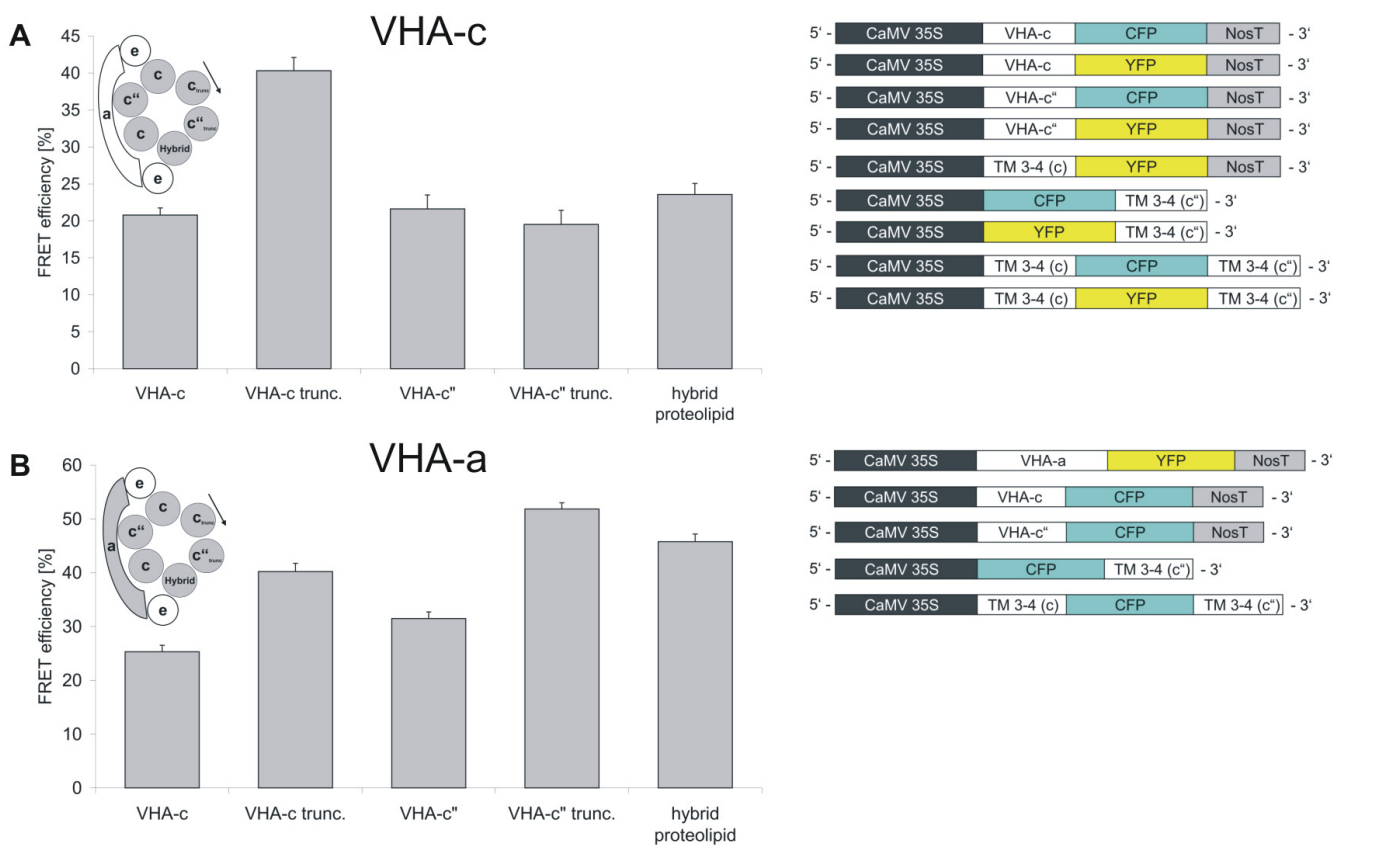
**FRET-efficiency between various proteolipid constructs and VHA-c (A) or VHA-a (B), respectively**. *A. thaliana *mesophyll protoplasts were co-transfected with VHA-c-YFP (A) or VHA-a-YFP (B) and the denoted VHA-CFP fusion proteins and analysed for energy transfer by quantitative confocal microscopy. The data represent the mean average ± SE (**A: **VHA-c: n = 80; VHA-c": n = 109; VHA-c_trunc_.: n = 78; VHA-c"_trunc_. n = 63; hybrid proteolipid: n = 88; **B: **VHA-c: n = 142; VHA-c": n = 117; VHA-c_trunc_.: n = 86; VHA-c"_trunc_.: n = 110; hybrid proteolipid: n = 90).

The relationship between structure and assembly was studied using FRET efficiency determinations between VHA-c fused to YFP as acceptor and the proteolipid constructs fused to CFP as donor. The latter set of contructs consisted of the full length VHA-c"1, the truncated variants VHA-c_trunc _and VHA-c"_trunc _as well as the hybrid proteolipid containing helices 3 and 4 each of VHA-c3 and VHA-c"1. In this approach no significant difference could be observed for VHA-c3, VHA-c"1, VHA-c"1_trunc _and the hybrid proteolipid subunit (T-test: p = 0.16–0.18). The FRET efficiencies ranged between 20–23%, whereas the incorporation of VHA-c_trunc _increased the FRET efficiency to very high 40.3% (Fig. 5A, p < 0.001). A similar effect was observed upon introducing VHA-c"-YFP (46.2%) and VHA-a-YFP (40.23%, p < 0.001) as acceptor partners for VHA-c_trunc _fused to CFP [see Additional files 1, 2, 3].

### FRET between proteolipid subunits-CFP-fusions and VHA-a-YFP

The next set of experiments addressed the structural interrelation between VHA-a and the proteolipid subunits. VHA-c3 and VHA-c"1 yielded similar FRET efficiencies in the range of 25–32% with VHA-a. However, the datasets differed slightly according to T-test evaluation (p = 0.026). The FRET efficiency value increased strongly between VHA-a and VHA-c_trunc_, VHA-c"_trunc _and the hybrid form, respectively, with FRET values in the range of 45.8 to 51.8% (Fig. 5B, p < 0.001). VHA-c"_trunc _is characterised by the absence of a functional glutamate residue, while the hybrid lacks a functional glutamate at helix 4 [see Additional files 1, 2, 3].

### FRET efficiency between VHA-e-CFP and the acceptors VHA-a-YFP, VHA-c-YFP and VHA-c"-YFP

**Figure 6:**
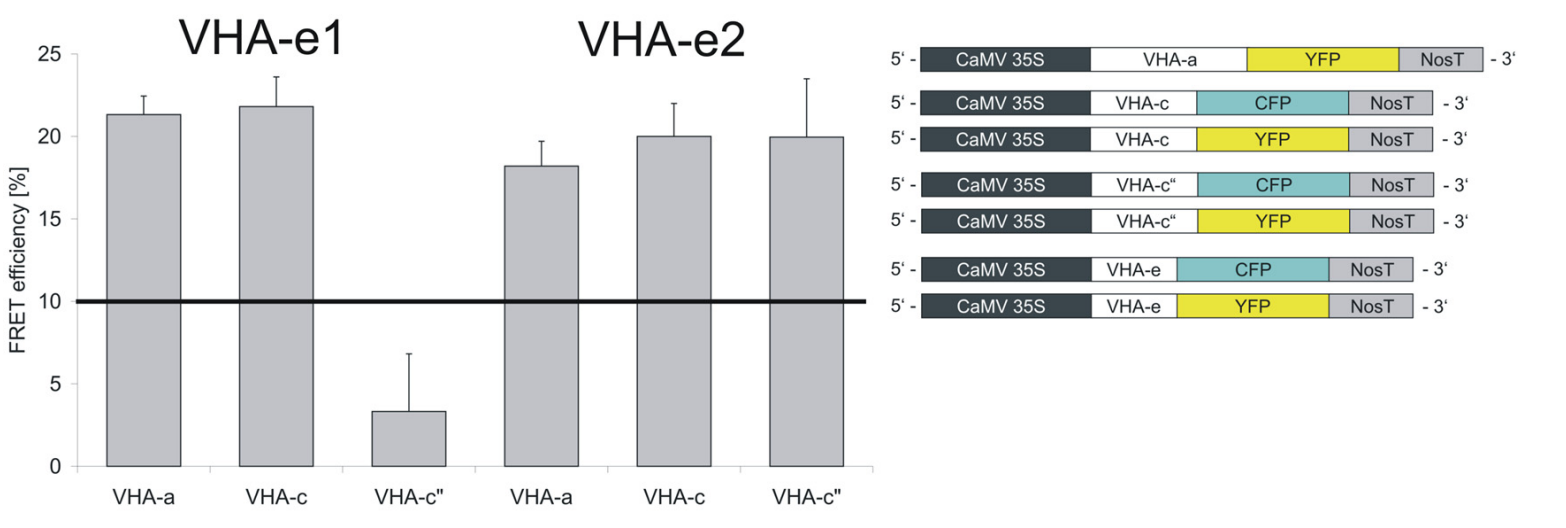
**FRET-efficiency between VHA-e isoforms and subunits VHA-a, VHA-c and VHA-c"**. CFP-fusion proteins of VHA-e1 and VHA-e2 were expressed in *A. thaliana *mesophyll protoplasts in combination with YFP-fusion proteins of VHA-a, VHA-c and VHA-c". FRET analysis was performed by confocal laser scanning microscopy (CLSM) to gain information on isoform-specific V-ATPase assembly. The data represent the mean average ± SE (**VHA-e1**: VHA-a: n = 62; VHA-c: n = 86; VHA-c": n = 40; **VHA-e2**: VHA-a: n = 36; VHA-c: n = 60; VHA-c": n = 52).

Both VHA-e isoforms were characterised by an energy transfer of approximately 20% relative to VHA-a and VHA-c. No significant difference was observed for VHA-e1 and VHA-e2 (p < 0.001). Only VHA-c" showed a specifity for VHA-e2 (20%) while VHA-e1 revealed marginal efficiency (3.3%) consistent with its distinct subcellular localisation (Fig. 6, p = 0.001) [see Additional files 1, 2, 3].

### FRET efficiency between VHA-a-YFP and VHA-A-CFP

FRET is occasionally considered as sensitive to artefacts. To address this important issue subsequent experiments investigated (i) the assembly of V_0 _and V_1 _subunits to a complete V-ATPase complex and (ii) potential artefacts as a consequence of overexpression, which should fail to associate in V_0_-V_1 _complexes. Protoplasts were co-transfected exemplarily with VHA-a-YFP and VHA-A-CFP. The FRET-efficiency between both labelled subunits was 44.8 ± 1.4 (mean ± SE, n = 96), hence demonstrating the efficient association of the subsectors V_1 _and V_0 _each bearing fluorophore fusions.

## Discussion

The presented experiments on subcellular localisation and quantitative *in vivo *FRET were performed to gain new insight in the function and isoform specifity of the proteolipid subunit VHA-c" and the membrane integral subunit VHA-e. In addition the FRET-measurements addressed the stoichiometry of VHA-subunits and the relative distances of the labeled termini. The energy transfer observed between CFP-labeled VHA-e1, VHA-e2, VHA-c3, VHA-c" and YFP-labeled VHA-a proved that the complete V_0_-complex is formed under conditions of fusion protein overexpression. This conclusion is supported by the observed energy transfer between multiple combinations of differently labeled proteolipids as well as between proteolipids and VHA-e. Functional incorporation of VHA-fusion proteins in the complex has been demonstrated before: (i) Without losing their function transgenic *Arabidopsis thaliana *were complemented with GFP-tagged VHA-a isoforms [30]. (ii) Co-expression of VHA-a-YFP and VHA-c-CFP in rice protoplasts displayed an increase in FRET-efficiency in response to salinity in a salt sensitive line but not in the salt tolerant line Pokkali. Hence, these results demonstrate structural implication of salinity by decreasing the proteolipid ring diameter. (iii) The obvious structural alterations were accompanied by increased vacuolar acidification as expected for a decreased H^+^/ATP ratio [33]. Furthermore, VHA-a as well as VHA-e were reported to require fully assembled V_0_-complexes to exit the ER. Unassembled VHA-a gets rapidly degraded in yeast [26,34-37]. In addition, the highly hydrophobic subunit VHA-e is postulated to associate with both the VHA-a and the proteolipid ring [26]. Except of the peripheral subunits VHA-C and VHA-H, which were demonstrated to be not essential for assembly in yeast [38], overexpression of individual V_1_-subunits results mainly in a destabilised and rapidly degraded fraction of excessively formed proteins similar to VHA-a. This was demonstrated for plant, yeast and insect V-ATPase subunits [31,39,40]. All these independent approaches confirm the validity of our approach since the modified V_0_-sectors associate with the V_1_-subsectors as demonstrated by the efficient energy transfer between VHA-A-CFP and VHA-a-YFP. Although the ratio of endogenous subunits and their corresponding fluorescent fusion proteins was not determined, the observed rates of energy transfer demonstrate the coexistence of CFP- and YFP-labeled subunits within single complexes. The absence of VHA-e and VHA-c" at the tonoplast is in agreement with published data on purified tonoplast proteins isolated and analyzed by mass spectrometry from tobacco [41] or *A. thaliana *[30,42]. Most VHA-polypeptides including the hydrophobic VHA-a and VHA-c were identified in the tonoplast fraction, while VHA-c" and VHA-e were undetectable. The reticulate distribution of VHA-c" and VHA-e2 resembles the pattern which was reported previously for the ER-network and associated Golgi-bodies [43]. In contrast, VHA-e1 was found in bodies which appeared only partially to be associated with the ER. The structures resembled the subcellular localisation of the TGN-specific VHA-a1 isoform [30]. In agreement with the different subcellular localisation, energy transfer was not observed between the FRET pairs VHA-c"-CFP and VHA-e1-YFP. Since the localisation of VHA-c" in plants also resembles the localisation of VHA-c" in mice [21], the ER and Golgi-specificity of VHA-c" might be a general feature of VHA-c" in (higher) eukaryotes.

Misfolded proteins of the secretory pathway often accumulate in the ER. This mechanism is referred to as unfolded protein response (UPR) [44]. It is rather unlikely that this process is causially responsible for the subcellular distribution observed for VHA-c" and VHA-e2. Besides the truncated VHA-c", the truncated VHA-c-YFP as well as the hybrid proteolipid subunit represent highly artificial proteolipid subunits that probably are strongly affected in their function. Both were not retained in the ER but were directed to the tonoplast. Overexpression of the ER- and Golgi-specific VHA-a isoform Stv1p resulted in at least partial replacement of the vacuolar isoform Vph1p in yeast, but not in retaining at the ER [45]. A similar effect cannot be excluded for the plant isoforms but also cannot provide an explanation for the exclusive localisation of VHA-c" and VHA-e in the ER but also the Golgi. The proteolipid subunits of V-ATPase are closely related to subunit c of the F-ATPsynthase. However during evolution, VHA-c underwent a gene duplication event with fusion of the coding regions likely generating an intermediate form with two catalytic glutamate residues [16]. While VHA-c lost the N-terminal one of these two catalytic glutamate residues, VHA-c" still is characterised by the presence of two conserved glutamate residues located in the helices 3 and 5 [20]. Both helices are equivalent to TM2 of the F-ATPase subunit c, but only the Glu of TM3 is functional [14]. Thus, VHA-c" interacts and can be crosslinked with VHA-a via TM3 [46]. In contrast to the structural relationship between VHA-c" and the ATPsynthase subunit c and thus between VHA-c and VHA-c", a cytosolic orientation of the VHA-c" N-terminus and the presence of 5 transmembrane regions was reported [19]. Aviezar-Hagai et al. [18] as well as Kawasaki-Nishi et al. [2] provided evidence for the presence of 4 transmembrane helices, with a cytosolic orientation of helix 1 as well as of the C-terminus. Recently, fusion proteins of VHA-c and VHA-c" were expressed in yeast strains disrupted in VHA-c". The VHA-c-c" construct complemented the *vma*^- ^phenotype in yeast [47]. The FRET-results of our work comply with the topology models of lumen-exposed C- and N-termini suggested by Flannery et al. [19] and Wang et al. [47] since very similar transfer efficiencies were obtained in our experiments involving VHA-c" and VHA-c, respectively. Additional support for this particular model derives from the incorporation of the hybrid proteolipid subunit into the complex, which appeared to be recognised as VHA-c-like. Surprisingly high FRET-efficiencies were detected between the hybrid protein and the truncated VHA-c", respectively, as donors and VHA-a as acceptor, whereas energy transfer between VHA-c and the truncated VHA-c" or the hybrid protein was similar to the energy transfer observed between VHA-c-CFP and VHA-c-YFP. The result can tentatively be explained by an increased probability for a confirmation where the proteolipid ring stays in a position with the truncated VHA-c" and the hybrid proteolipid subunit, respectively, residing next to VHA-a. This arrangement might be a steric effect caused by the position of the fluorophore at the N-terminus of VHA-c" helix 3 or by the lack of a catalytically active helix, which was demonstrated to alter its confirmation when it passes the VHA-a during the catalytic cycle [46]. In this scenario, the fluorophores fused to the proteolipid subunit and to VHA-a, respectively, are localised in close vicinity at the proteolipid/VHA-a interface leading to the observed increase in FRET efficiency. In contrast, the truncated VHA-c-CFP showed a high energy transfer with VHA-c-YFP and VHA-a-YFP as acceptor, respectively. This demonstrates the reduction of the average distances between the proteolipid subunits and between VHA-a and the truncated VHA-c and is suggested to indicate the reduction of the proteolipid ring diameter by the replacement of the native proteolipids with 4 by truncated subunits with only 2 transmembrane helices. In a simplified calculation, incorporation of each molecule of truncated VHA-c instead of VHA-c may decrease the circumference by 1/12th part due to the lack of two transmembrane helices out of 24. Assuming a proteolipid ring diameter of initially 6.6 nm, an increase of FRET-efficiency from approximately 20% to 40% would correspond to the replacement of two subunits VHA-c by the truncated VHA-c. Acclimatory alterations of the proteolipid ring diameter in response to salinity were reported before for the V-ATPase of *Oryza sativa *[33] and *Mesembryanthemum crystallinum*, but postulated to be caused by a reduced number of proteolipids [48].

Gibson et al. [20] suggested a dimerization of VHA-c" in yeast whereas Powell et al. [49] could identify only one copy of VHA-c" within the VHA-complex. Our results of low FRET efficiency between VHA-c"-CFP and VHA-c"-YFP and the absence of YFP-fluorescence in VHA-c"-splitYFP approaches indicate the presence of monomeric VHA-c". The afore reported dimerization might be caused by V_0 _*trans *complexes or fusion pore formation as postulated by Peters et al. [8] and thus represents a close arrangement of two proteolipid rings. The FRET-pair VHA-c"-CFP and VHA-e2-YFP may be taken as a negative control as both subunits are located in distinct complexes. The slightly higher FRET-efficiency between VHA-c"-CFP and VHA-c"-YFP might be caused by temporary V_0 _*trans *complexes as well, but in this scenario the distance between the fluorophores would span two membranes and thus exceed 10 nm.

**Figure 7:**
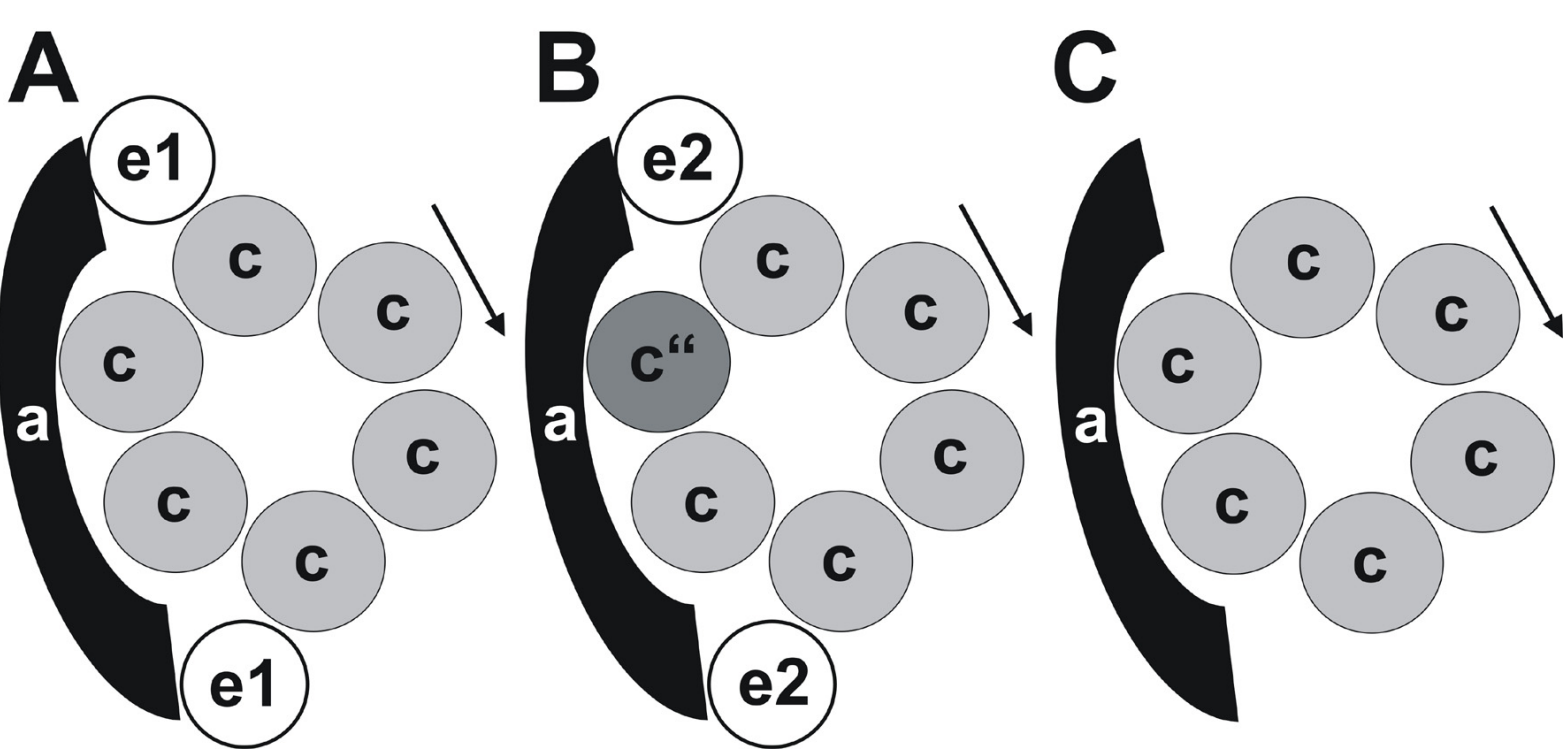
**Hypothetical scenario of organelle-specific assembly of subsector V_0_**. Based on the subcellular localization and the FRET-results three structurally distinct V_0_-sectors can be hypothesized. (A) The putative TGN-specific V_0_-sector comprises subunits VHA-a, VHA-c and VHA-e1, whereas the ER-specific subsector contains VHA-a, VHA-c, VHA-c" and VHA-e2. (C) The vacuolar V-ATPase lacks VHA-c" and VHA-e.

The membrane integral subunit VHA-e is encoded by two isogenes in *A. thaliana*. The YFP-fusions VHA-e1-YFP and VHA-e2-YFP differed in their subcellular localisation but both were absent from the tonoplast. Since the V-ATPase is active in the vacuolar membrane, VHA-e is not essential for proton pumping activity. Based on the VHA-e isoforms, three types of V-ATPases are suggested to be formed, which differ in their subcellular localisation (Fig. 7). (i) V-ATPases containing VHA-e1 are localised to the putative TGN. (ii) V-ATPase containing VHA-e2 (and VHA-c") remain in the ER and (iii) V-ATPases lacking both VHA-e isoforms can be found at the tonoplast. These findings suggest a role of VHA-e in V-ATPase sorting along the secretory pathway rather than in catalysis. In addition, more than one copy of VHA-e was identified within the complex by FRET independent of the isoforms. Up to now, VHA-e was reported to be monomeric [25,26]. The FRET-efficiency was approximately 20% for the FRET pairs VHA-e-CFP/VHA-a-YFP and VHA-e-CFP/VHA-c-YFP. Thus, VHA-e seems to reside next to the interface of VHA-a and VHA-c.

## Conclusion

Taken together, VHA-c" is a single copy subunit within the V-ATPase complex and localises to the ER and Golgi, but is absent from the tonoplast. Its structure resembles that of VHA-c with the exception of the catalytically essential glutamate residue, which is localised in the second helix instead in the fourth. The plant V_0_-sector was identified as highly flexible complex, which tolerates the incorporation of truncated as well as hybrid proteolipid subunits. The structural flexibility may be the basis of a long term regulation of V-ATPase activity by alterations of the proteolipid ring diameter, allowing the adaptation to environmental stress conditions like salinity by tuning transport capacity and coupling ratio [50]. The subunit VHA-e consists of two membrane integral helices and is incorporated into the VHA-complex with more than one copy. The VHA-e isoforms in *A. thaliana *showed a distinct subcellular localisation, however, both were absent from the tonoplast like VHA-c" confirming previous proteomics studies [41,42]. Since the V-ATPase is active at the tonoplast, VHA-c" as well as VHA-e isoforms can not be essential for proton transport in plant V-ATPase. It should be noted that although regulatory modulation of the V-ATPase activity by VHA-c" and VHA-e can not be fully excluded, it seems more likely that these subunits are involved in organelle-specific assembly or sorting of V-ATPases along the secretory pathway. The ER-specific V-ATPase complexes contain VHA-c" and VHA-e2, while the putative TGN-specific complex contains VHA-e1 and the vacuolar complex neither VHA-c" nor VHA-e.

## Methods

### Materials

*A. thaliana *(Columbia) was grown in soil-culture in a growth chamber with 12 h light (240 μmol quanta m^-2 ^s^-1^, 19°C) and 12 h dark (18°C) with 60% relative humidity. For protoplast isolation *A. thaliana *leaves were harvested from soil grown plants, and for mRNA isolation, from hydroponic cultures each at the age of about four weeks.

### Molecular biology

The full length cDNA sequences of VHA-c3 (At4g38920), VHA-c"1 (At4g32530), VHA-c"2 (At2g25610), VHA-e1 (At5g55290), VHA-e2 (At4g26710) and VHA-A (At1g78900) from *A. thaliana *were obtained from cDNA-libraries. The open reading frames were amplified without the terminating stop codon by PCR with forward and reverse primers introducing BamHI and AgeI restriction sites at the 5' and the 3' end, respectively. Following restriction, the PCR-product was cloned into the vectors 35S-CFP-NosT and 35S-YFP-NosT [31]. The construct VHA-a-YFP was based on the *Mesembryanthemum crystallinum *VHA-a subunit [29]. A fragment of 35S-CFP and 35S-YFP spanning from the BsrGI to the NdeI restriction site was amplified by PCR to substitute the codon CAA for the *Ochre *Stop codon. The fluorescent protein open reading frame was replaced by the amplified fragment and the resulting vectors were denominated 35S-CFP-C and 35S-YFP-C, respectively. A chimeric construct was achieved by amplifying the open reading frame of VHA-c aa 80–164 and introducing flanking 5' BamH I and 3' Age I restriction sites. On the other hand, the open reading frame of VHA-c"1 aa 88–180 was amplified with flanking 5' Not I and 3' EcoR I restriction sites. Both fragments were introduced into 35S-CFP-C and 35S-YFP-C, respectively. Truncated proteolipid variants VHA-c_trunc _and VHA-c"_trunc _were intermediate constructs of hybrid formation and were included in the analysis.

Using AgeI and BsrG1-restriction sites the EYFP-gene in 35S-YFP-NosT was replaced by the N-terminal (encoding aminoacids 1–155) and C-terminal (aminoacids 156–238) open reading frame fragments of EYFP, respectively. The resulting split-YFP vectors were restricted by BamHI and AgeI for ligation with the open reading frame of VHA-c" isoform 1, VHA-c, VHA-e1 and VHA-e2 [see Additional file 4].

### *In silico *analysis

The amino acid sequences of VHA-c" from *A. thaliana *(NP_194979; NP_180132;) and *Saccharomyces cerevisiae *(P23968) as well as VHA-e sequences from *A. thaliana *(NP_568823, CAB79526), *Bos taurus *(CAA75570), *Manduca sexta *(CAA06822) and *S. cerevisiae *(NP_958835) were obtained from the NCBI database. The alignments were constructed with CLUSTAL W. Hydrophobic amino acids where predicted by the Expasy topology prediction tools , HMMTOP and PredictProtein. Sequences of VHA-c3 (P59227), VHA-c"1 (NP_194979) and VHA-e1 (NP_568823) were submitted and analysed for hydrophobicity.

### Analysis of the transcript level

The transcript levels of the VHA-c" isoforms At4g32530 and At2g25630 and the five VHA-c isoforms (At4g34720, At1g19910, At4g38920, At1g75630, At2g16510) were assessed by semi-quantitative RT-PCR. The transcript accumulation was compared among leaf and root tissue as well as between the isoforms following normalization on similar amounts of actin. The amplification of the transcripts required 32 cycles. RT-PCRs were performed with H_2_O instead of cDNA as negative control. The used primers were intron-spanning, aberrant amplification of genomic DNA results in significant increase of product length (Table 1). The resulting images of ethidiumbromide stained gels were quantified by Gelscan software, and the transcript levels related to the strongest signal within each set of three independent experiments.

**Table 1:** Primer sequences and resulting product size of RT-PCR with genomic DNA and cDNA as template

	**Primer Sequences**	**genomic DNA [bp]**	**cDNA [bp]**
c"1 (At4g32530)	**F: **AGAGAGAGAGAGAGAGAG	1578	561
	**R: **CTATTTTGTAGGCCATGT		
c"2 (At2g25610)	**F: **TCAAATTCATTCATATCA	943	570
	**R: **CTATTTTGTAGGCCATGT		
c1 (At4g34720)	**F: **ATTTCAGATTTAAGATCT	979	531
	**R: **TCATTCGGCTCTGGACTGACC		
c2 (At1g19910)	**F: **GTCTCATTCCCGATCAGA	1039	532
	**R: **TCATTCGGCTCTGGACTGACC		
c3 (At4g38920)	**F: **AGCACATATTTTAAGATC	1617	528
	**R: **TCATTCGGCTCTGGACTGACC		
c4 (At1g75630)	**F: **ATCTCATCGGAGCAACAG	1402	532
	**R: **TCATTCGGCTCTGGACTGACC		
c5 (At2g16510)	**F: **ATCCAAAACTTTGAGATC	829	519
	**R: **TCATTCGGCTCTGGACTGACC		

### Isolation and transfection of mesophyll protoplasts

The isolation and PEG-mediated transfection of *A. thaliana *leaf mesophyll protoplasts was performed as described before [29].

### Confocal laser scanning microscopy

For confocal laser scanning microscopy a Leica TCS SP2 confocal system with 63-fold magnification (oil immersion objective, NA = 1.4) and the double dichroic filterset DD458/514 was used. The scan speed was 400 Hz, the image resolution 1024*1024 pixels and background noise was eliminated by line averaging and a PMT offset value of -20. CFP and chlorophyll were excited by the 458 nm and YFP by the 514 nm laserline. CFP emission was detected in the range of 470–510 nm, FRET and YFP in the range of 530–600 nm and autofluorescence between 650 and 700 nm.

### Investigation of subcellular localisation

Localisation experiments of fusion proteins were performed in *A. thaliana *mesophyll protoplasts. The signal amplification was adjusted for each fluorophore independently. The line-averaging was 4 and the pixel brightness was encoded by 8 bit. Fluorescent proteins are shown in green, plastidic autofluorescence in red. [31].

### FRET measurements

The transfer efficiency between the FRET-pair CFP and YFP was measured within mesophyll protoplasts by sensitized acceptor emission [31]. CFP was detected in the range of 470–510 nm (CFP-intensity I_CFP_) and FRET (FRET raw intensity I_FRET_) in the range of 530–600 nm after 458 nm excitation. The YFP-reference was obtained in the range of 530–600 nm after 514 nm excitation (YFP-intensity I_YFP_). The line-averaging was 2 and the pixel brightness was encoded by 12 bit to improve the signal to noise ratio. The signal amplification was identical for CFP and YFP-detection. The detection of YFP direct excitation by the 458 nm laserline depended on the intensity of the 514 nm laserline (for correction see data processing) [see Additional files 2 and 3].

### Data processing

The FRET images were quantified using the Quantify (profile) modul of the Leica confocal software (Freeware LCS Lite). Maxima were read out and FRET efficiency was calculated. The correction factors k_dir _for YFP direct excitation were 1.027 (5% laser intensity), 0.452 (7%), 0.084 (10%) and 0.058 (12%). Direct excitation intensities of YFP and CFP-crosstalk were subtracted from the signal recorded in the FRET-channel. The remaining intensity of the FRET channel was finally normalized to the sum of the emission intensities in the donor channel and the FRET channel:

(1) E = (I_FRET _- 0.61 * I_CFP _- k_dir _* I_YFP_)/(I_FRET _+ I_CFP_).

Arithmetic means as well as standard errors were calculated. Data sets were analyzed for significance of difference by Student's T-test.

### Bimolecular fluorescence complementation

The formation of the YFP-fluorophor was detected in the range of 530–600 nm upon 514 nm excitation. CFP-expression under control of the CaMV35S promoter served as standard for the expression level of the cell. The CFP-emission was recorded between 470 and 510 nm with 458 nm excitation. The YFP-fragments were fused to the C-terminus of VHA-c" in order to detect VHA-c" dimerization. Cells transfected with CaMV35S-CFP-NosT were used as background control, cells transfected with CaMV35S-YFP-C-terminus and CaMV35S-YFP-N-terminus served as control for cytosolic formation of the YFP-fluorophore when no putative interaction partners were fused to the fragments. The YFP-expression was normalised to the observed CFP-expression.

## List of abbreviations

The abbreviations used are: CFP: cyan fluorescent protein; YFP: yellow fluorescent protein; FRET: fluorescence resonance energy transfer; VHA: vacuolar proton translocating ATPase; TGN: *trans *Golgi network; TM: transmembrane helix.

## Authors' contributions

TS carried out protoplast transfections, *in silico *sequence analysis and confocal microscopy, DS performed transcript analysis, DG participated in design of the study, MS participated in FRET analysis and calculation, K–JD conceived the study, participated in its design and coordination. All authors read and approved the manuscript.

## Supplementary Material

Additional file 1 FRET-measurements. The following FRET-efficiencies were measured and displayed in a model of the subsector V_0_: A) FRET-efficiencies between VHA-e isoforms  and VHA-a, VHA-c and VHA-c", respectively. B) FRET-efficiencies between VHA-a and proteolipid subunits as well as between VHA-c and proteolipid subunits. C) Substitution of two VHA-c by two truncated VHA-c results in a reduced proteolipid ring diameter and hence in increased FRET-efficiency. Click here for file

Additional file 2 FRET images. FRET images were selected, which appear representative for low FRET-efficiency (<10%), medium FRET efficiency (~20%) and high FRET efficiency (>40%). CFP and YFP denominate the reference channels. "raw FRET" displays the recorded emission in the FRET channel without correction for CFP-crosstalk and YFP-direct excitation. CFP-crosstalk and YFP-direct excitation are considered in "corr. FRET". Click here for file

Additional file 3 FRET efficiencies. The table contains the observed FRET-efficiencies. The mean average ± SE is shown. Click here for file

Additional file 4 List of constructs. Click here for file
