# Supplementary figures and images for: Organelle-specific isoenzymes of plant V-ATPase as revealed by in vivo-FRET analysis
Source: BMC Cell Biol. 2008 May 28;9:28. doi: 10.1186/1471-2121-9-28 (PMC2424043; doi:10.1186/1471-2121-9-28)

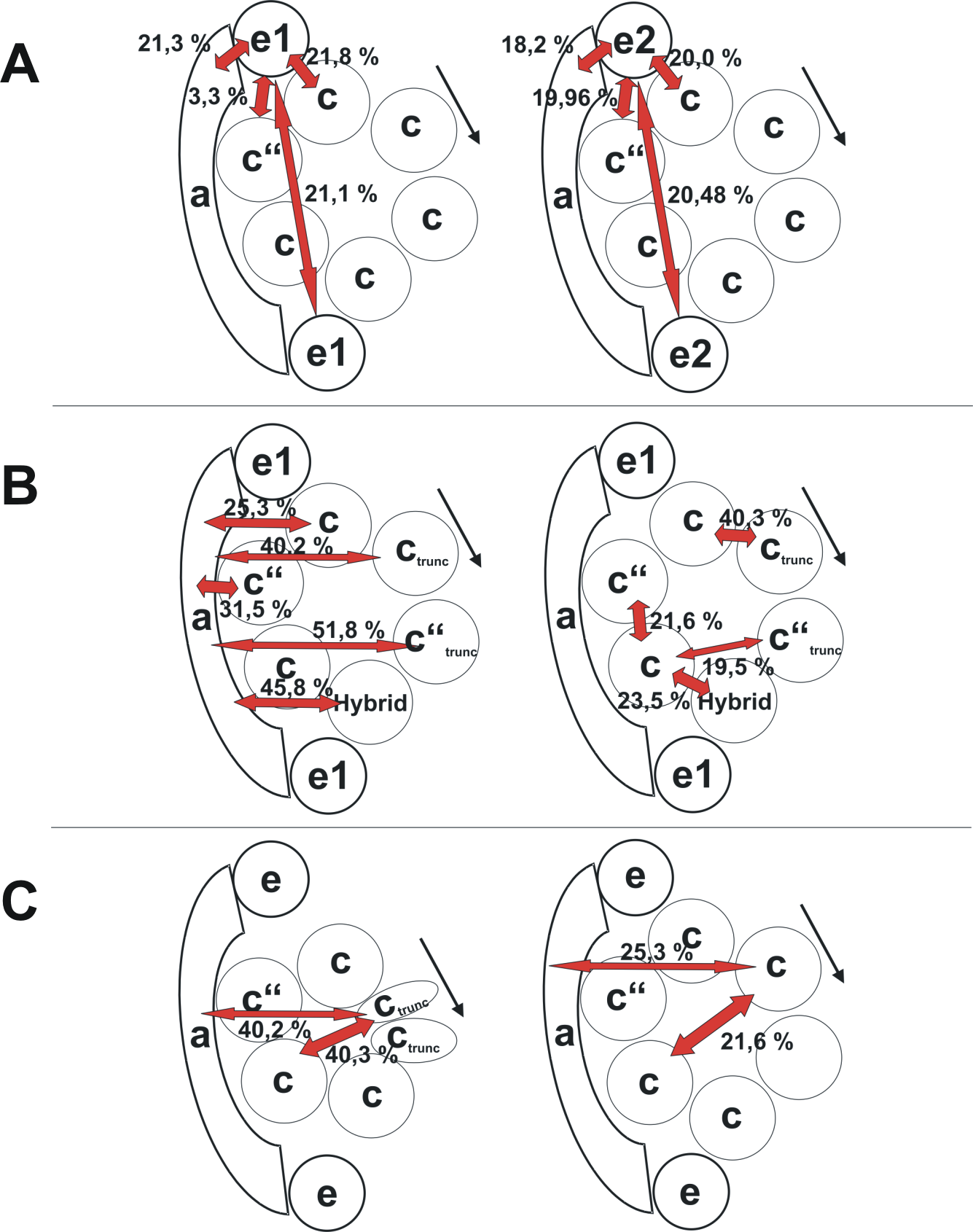

Supplement: Additional file 1 — FRET-measurements. The following FRET-efficiencies were measured and displayed in a model of the subsector V0: A) FRET-efficiencies between VHA-e isoforms and VHA-a, VHA-c and VHA-c", respectively. B) FRET-efficiencies between VHA-a and proteolipid subunits as well as between VHA-c and proteolipid subunits. C) Substitution of two VHA-c by two truncated VHA-c results in a reduced proteolipid ring diameter and hence in increased FRET-efficiency. [file 1471-2121-9-28-S1.tiff]

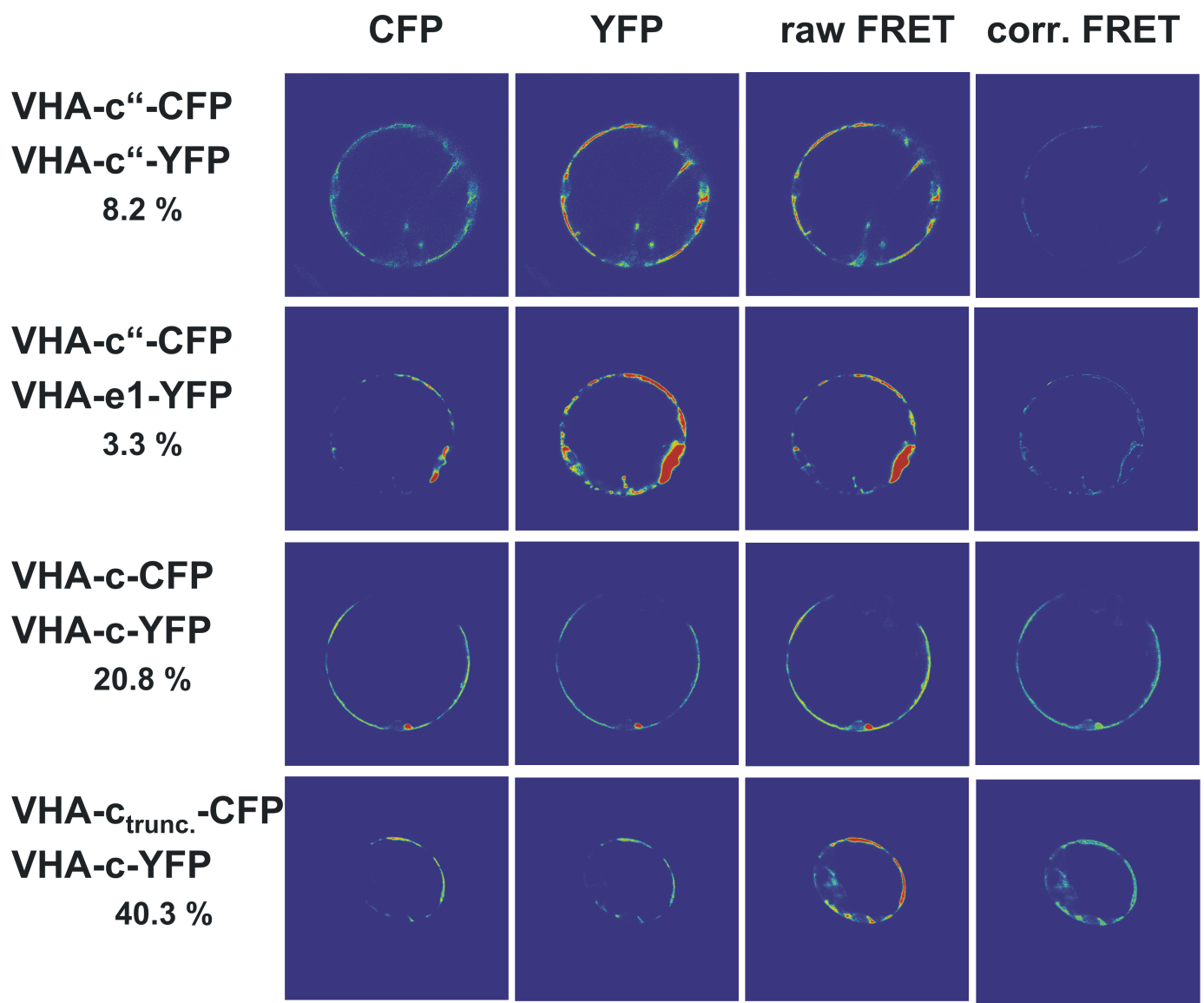

Supplement: Additional file 2 — FRET images. FRET images were selected, which appear representative for low FRET-efficiency (<10%), medium FRET efficiency (~20%) and high FRET efficiency (>40%). CFP and YFP denominate the reference channels. "raw FRET" displays the recorded emission in the FRET channel without correction for CFP-crosstalk and YFP-direct excitation. CFP-crosstalk and YFP-direct excitation are considered in "corr. FRET". [file 1471-2121-9-28-S2.tiff]

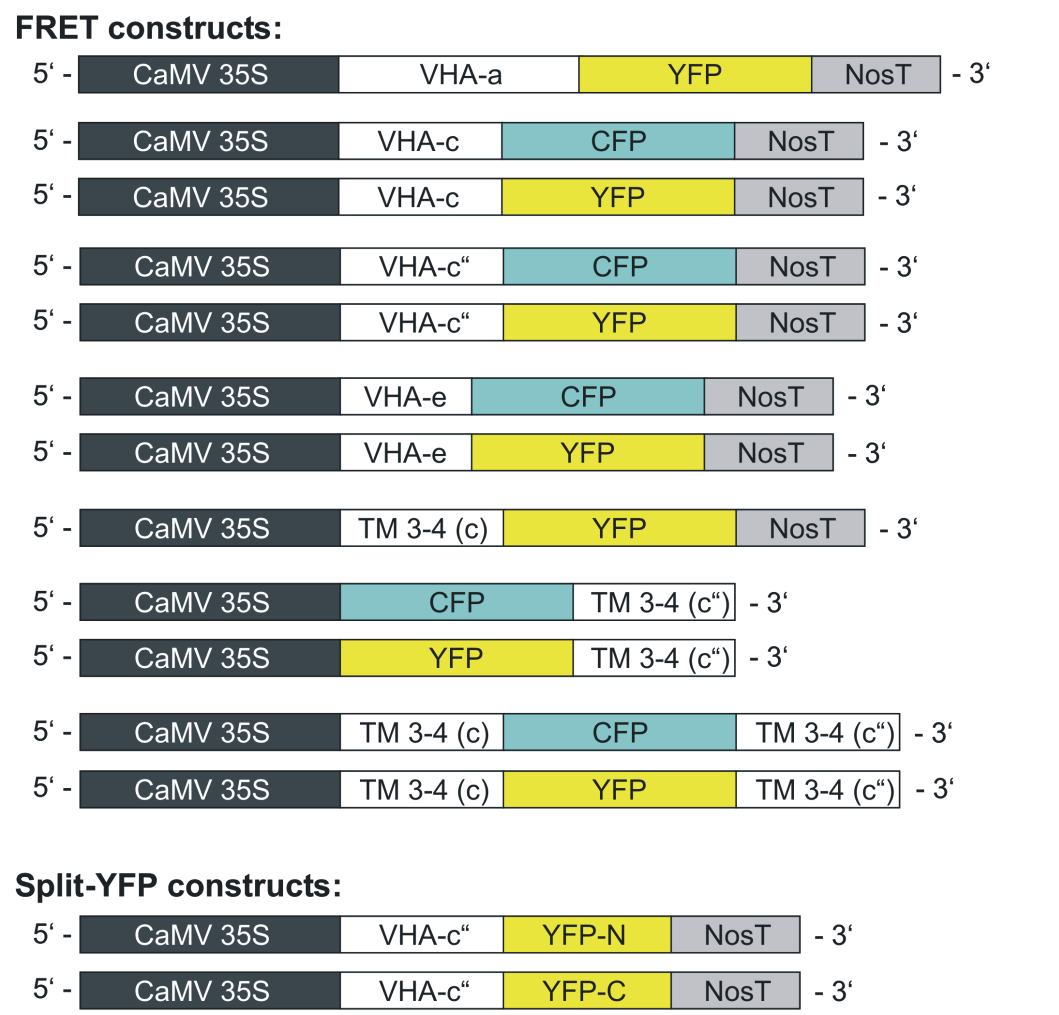

Supplement: Additional file 4 — List of constructs. [file 1471-2121-9-28-S4.tiff]
