# Supplementary material for: Organelle-specific isoenzymes of plant V-ATPase as revealed by in vivo-FRET analysis
Source: BMC Cell Biol. 2008 May 28;9:28. doi: 10.1186/1471-2121-9-28 (PMC2424043; doi:10.1186/1471-2121-9-28)
Supplement: Additional file 3 — FRET efficiencies. The table contains the observed FRET-efficiencies. The mean average ± SE is shown. [file 1471-2121-9-28-S3.pdf]

	VHA-a	VHA-c	VHA-c”	VHA-e1	VHA-e2	VHA-c_{trunc}	VHA-c”$_{trunc}$	Hybrid
**VHA-a**		25.3 ± 1.2<br>n=142	31.5 ± 1.2<br>n=117	21.3 ± 1.1<br>n=62	18.2 ± 1.5<br>n=36	40.2 ± 1.5<br>n=86	51.8 ± 1.2<br>n=110	45.8 ± 1.4<br>n=90
**VHA-c**	25.3 ± 1.2<br>n=142	20.8 ± 1.0<br>n=80	21.6 ± 1.8<br>n=109	21.8 ± 1.8<br>n=86	20.1 ± 1.9<br>n=53	40.3 ± 1.9<br>n=78	19.5 ± 1.9<br>n=63	23.6 ± 1.5<br>n=88
**VHA-c”**	31.5 ± 1.2<br>n=117	21.6 ± 1.8<br>n=109	8.2 ± 1.7<br>n=71	3.3 ± 3.5<br>n=40	20.0 ± 3.5<br>n=30		14.8 ± 1.5<br>n=59	
**VHA-e1**	21.3 ± 1.1<br>n=62	21.8 ± 1.8<br>n=86	3.3 ± 3.5<br>n=40	21.1 ± 2.0<br>n=85	20.5 ± 1.8<br>n=78			
**VHA-e2**	18.2 ± 1.5<br>n=36	20.1 ± 1.9<br>n=53	20.0 ± 3.5<br>n=30	20.5 ± 1.8<br>n=78	28.8 ± 2.4<br>n=50			
**VHA-c_{trunc}**	40.2 ± 1.5<br>n=86	40.3 ± 1.9<br>n=78						
**VHA-c”$_{trunc}$**	51.8 ± 1.2<br>n=110	19.5 ± 1.9<br>n=63	14.8 ± 1.5<br>n=59					
**Hybrid**	45.8 ± 1.4<br>n=90	23.6 ± 1.5<br>n=88						20.5 ± 1.7<br>n=121
